# Enabling Fine Sample Rate Settings in DSOs with Time-Interleaved ADCs

**DOI:** 10.3390/s22010234

**Published:** 2021-12-29

**Authors:** Mauro D’Arco, Ettore Napoli, Efstratios Zacharelos, Leopoldo Angrisani, Antonio Giuseppe Maria Strollo

**Affiliations:** Department of Electric and Information Technology Engineering (DIETI), University of Napoli Federico II, Via Claudio 21, 80125 Napoli, Italy; etnapoli@unina.it (E.N.); efstratios.zacharelos@unina.it (E.Z.); angrisan@unina.it (L.A.); astrollo@unina.it (A.G.M.S.)

**Keywords:** digital storage oscilloscope, time-interleaving, time-base

## Abstract

The time-base used by digital storage oscilloscopes allows limited selections of the sample rate, namely constrained to a few integer submultiples of the maximum sample rate. This limitation offers the advantage of simplifying the data transfer from the analog-to-digital converter to the acquisition memory, and of assuring stability performances, expressed in terms of absolute jitter, that are independent of the chosen sample rate. On the counterpart, it prevents an optimal usage of the memory resources of the oscilloscope and compels to post processing operations in several applications. A time-base that allows selecting the sample rate with very fine frequency resolution, in particular as a rational submultiple of the maximum rate, is proposed. The proposal addresses the oscilloscopes with time-interleaved converters, that require a dedicated and multifaceted approach with respect to architectures where a single monolithic converter is in charge of signal digitization. The proposed time-base allows selecting with fine frequency resolution sample rate values up to 200 GHz and beyond, still assuring jitter performances independent of the sample rate selection.

## 1. Introduction

The possibility of different sample rate choices in digital storage oscilloscopes (DSOs) is typically implemented by means of a digital circuit, positioned between the analog to digital converter (ADC) and the acquisition memory, that can seamlessly decimate the digitized signal by an integer factor [1,2,3]. Here, decimation has to be intended in a wide sense as selecting one out of *M* consecutive samples that are returned by the ADC, where *M* is the decimation factor [4,5]. This ingenious approach lets the ADC steady operate at a fixed-frequency, which is chosen equal to the maximum frequency for which it has been designed, and assures stability performances, expressed in terms of absolute jitter, that are independent of the chosen sample rate [6,7,8]. Moreover, it simplifies the data transfer from the ADC to the acquisition memory, because the memory access rate is always equal to a submultiple of a fixed frequency, namely the maximum sample rate [9,10]. On the counterpart, this approach substantially limits the possible choices of the sample rate, that, due to additional design constraints, fall upon three values per decade: typically either {2,5,10} or {2,4,10} [11,12,13]. Consequently, one has poor control of the time interval capture in waveform analysis, and less possibilities for an optimal usage of the memory resources [14,15]. For instance, one can not set coherent sampling conditions in the analysis of steady alternate waveforms, as well as optimal frequency span and resolution in FFT analyses [16,17,18,19].

A time base system that allows selecting the sample rate with very fine frequency resolution has recently been proposed in [20,21]. This solution improves the digital circuit deployed between the ADC and the acquisition memory such that it can digitally downsample the signal by a factor within the interval $\left(\frac{1}{2},1\right)$. This is sufficient to grant fine sample rate choices in a wide range, since fine frequency resolution becomes also available to lower values using one decimation factor within the interval $\left(\frac{1}{2},1\right)$ followed by an additional integer decimation step [22,23,24]. Clearly, the need of real-time execution and fine sample rate selections, make the downsampling task much more challenging with respect to post-processing applications, where downsampling can be considered little more than an elementary task [25,26,27,28,29]. A physical synthesis of the digital circuit shows that such a time base is capable of operating on signals at a maximum sample rate slightly above 5 GHz [21].

In the presence of sample rates that reach 200 GHz and above, as those available from DSOs that exploit several time-interleaved ADCs, the time base system developed for a single channel architecture cannot be straightforwardly adapted to cope with parallel architectures [30,31,32]. In time-interleaved structures, *L* parallel channels, each one hosting an individual ADC, acquire the input signal at the same rate, but with a time-offset equal to $\frac{1}{L}$ of the sample period. As a result, the aggregate throughput is *L* times the sample rate of the individual ADCs. Time interleaved structures are complemented with a digital circuit that combines the sample streams and carries out further real-time operations to produce a calibrated response and/or enable different acquisition modes. The most common approaches exploit polyphase filters, which are suitable to manage parallel data streams [33,34,35]. Unfortunately, they lack however of adequate reconfigurability features to enable fine sample rate selection capabilities.

A dedicated solution to enable fine sample rate selections for DSOs with time-interleaved ADCs, which inherits some concepts of the one for single channels architectures and extends them to cope with parallel architectures is studied and proposed. More specifically, as for the pre-existing solution, the digital signal is filtered with a filter characterized by dynamically generated coefficients to obtain a version that contains both useful samples and dummy samples. At the acquisition stage the useful samples are retrieved while the dummy ones are discarded for achieving the reduced sample rate chosen by the user. However, the proposed solution parallel distributes the processing operations to cope with a stream of bunched samples, each bunch formed with the samples released by the parallel ADCs within the same clock period. This is enabled by means of some important novelties consisting in: (i) a parallel processing scheme for filtering the bunched signal with a filter characterized by dynamically varying coefficients; (ii) an original strategy for defragmenting each bunch and separating useful from dummy samples; (iii) a memory management approach for assembling constant size arrays that are either full of useful samples or of dummy samples, such that storing the first and discarding the latter allows acquiring the signal at the selected sample rate. It is worth underlining that the main challenge faced by the proposed solution is satisfying real-time execution requirements, which are due for all the aforementioned tasks, such that the user can rely on fine sample rate choices up to 200 GHz and beyond.

The article overviews the pre-existing solution, which is tailored for a DSO with a monolithic ADC, i.e., a single channel architecture, in Section 2, and presents the new solution, designed for a multi-channel architecture, in Section 3, where also some results related to a synthesis of the circuit to implement the proposed approach are given. Throughout the article the attention is mainly paid to illustrate methods and operation principles in the clearest possible way, preferring to this end some degree of abstraction in place of a faithful description of the layout of the digital circuit that implements the proposed solution.

## 2. Sample Rate Selection in a Single Channel Architecture

The fundamental idea underneath the resampling strategy for a single channel architecture can be explained considering a couple of clocks at different frequencies. The first clock is a physical clock with frequency $f_{ck}$, coinciding with the maximum sample rate offered by the system; this clock ticks the sampling instants providing the timing to the internal ADC. The second clock is a fictitious clock characterized by frequency $Cf_{ck}$, where C<1, that ticks the resampling instants.

**Figure 1:**
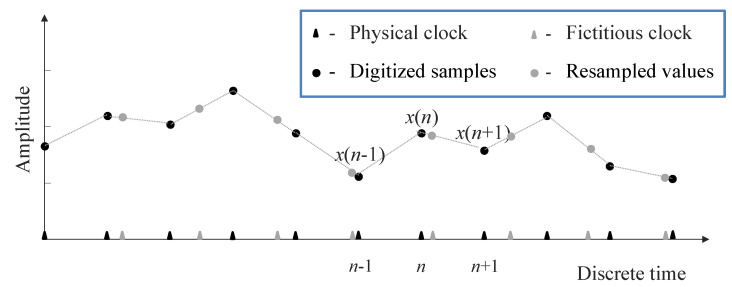
Samples produced by the ADC at the time instants ticked by the physical clock, and resampled values at time instants ticked by the fictitious clock.

As shown in Figure 1 the resampling instants generally fall between a couple of consecutive time instants ticked by the first clock. The resampling instants exhibit a delay with respect to a previous sampling instant and a lead with respect to the subsequent sampling instant. The amounts of both delay and lead are used to calculate the resampled value falling between the instants *n* and (n+1), named y(n), as weighted average of the samples x(n) and x(n+1). This strategy identifies a linear interpolation method that can be implemented provided that the lead and delay, namely the coefficients in the weighted average, are identifiable, as they are on the base of the value *C* implied by the sample rate chosen by the user.

More specifically, the value y(n) is determined by combining the samples x(n) and x(n+1) according to:

(1) $$y\left(n\right)=a\left(n\right)x\left(n\right)+\bar{a}\left(n\right)x\left(n+1\right)$$

where a(n) quantifies in $T_{ck}$ units ($T_{ck}$ is the reciprocal of $f_{ck}$), the time lead with respect to instant (n+1) and $\bar{a}\left(n\right)=1-a\left(n\right)$ the delay with respect to instant *n*. The coefficients are time varying and can be determined using a recursive formula: the coefficient a(n) is obtained by subtracting to its previous value the lead reduction caused by the longer period of the resampling clock, equal to $\frac{T_{ck}}{C}-T_{ck}$, which in $T_{ck}$ units equals $\frac{1-C}{C}$; the coefficient $\bar{a}\left(n\right)$ is in parallel obtained by adding to its previous value the same quantity, which represents the increment of the time delay of the resampling instant with respect to the previous sampling instant.

It is worth noticing that a negative lead and a delay greater than one contextually occur anytime two consecutive ticks by the fictitious clock include two consecutive ticks by the physical clock. In these cases the resampled value obtained with the weighted average is considered a dummy one. Dummy values are not stored in the acquisition memory, thus extracting a subsequence from the signal y(n); this subsequence represents the resampled version of the signal x(n) and it is characterized by a lower sample rate, namely $Cf_{ck}$, (longer period: $\frac{T_{ck}}{C}$). Also, the occurrence of dummy values can be detected by checking the values of a(n) or $\bar{a}\left(n\right)$, which by nature must be positive values within (0,1). The values of the coefficients that underflow or overflow (0,1) are wrapped at the next clock tick around 0 and 1 by adding and subtracting the unit value, respectively, to a(n) and $\bar{a}\left(n\right)$. The algorithm for computing the values of the coefficients at the *n*-th step can be described in terms of coded instruction as in Algorithm 1, where the condition a(n)<0 is used instead of the equivalent one, $\bar{a}\left(n\right)>1$ to recognize dummies occurrences; the coefficients are initialized as a(0)=1 and $\bar{a}\left(0\right)=0$.

**Algorithm** **1:** Algorithm for computing the weighting coefficients.if a(n)<0 then {
   a(n)=a(n−1)+1;
$\;\bar{a}\left(n\right)=\bar{a}\left(n-1\right)-1;$
} else
$\;a\left(n\right)=a\left(n-1\right)-\frac{1-C}{C};$
$\;\bar{a}\left(n\right)=\bar{a}\left(n-1\right)+\frac{1-C}{C};$
} end


Managing the storage process for skipping dummy samples and creating the lower rate stream is attained using a pointer to the acquisition memory (or the interface buffer); the pointer undergoes (unitary) self-increment by default, except when a negative a(n) is detected. The dummy samples are thus temporarily stored in the memory but immediately overwritten by the next sample. For example, with reference to Figure 1, at the discrete time instant *n* the weighted average of x(n−1) and x(n) is a dummy value, which is temporarily stored in memory, and overwritten at the next clock cycle by the resampled value that falls between the time instants *n* and n+1.

The described method uses a couple of dynamically generated coefficients to perform filtering and subsequently determine the resampled value. Filtering can be simply recognized to be a linear interpolation between adjacent samples, which corresponds to a filter with poor roll-off. Specifically, the frequency response of the filter, H(f), is analytically described by:

(2) $$H\left(f\right)=T_{ck}sinc^{2}\left(fT_{ck}\right)$$

The aliasing contributions produced by downsampling the digital signal is however sufficiently contrasted by the filter in Equation (2) anytime the output sample rate is much greater than the Nyquist rate of the input signal. In the practice, experts DSO users are aware of the convenience of oversampling the signal for collocating the alias contributions in the deep attenuation band of the anti-alias filter.

Also, the filter exhibits a lack of flatness in the pass-band that can be compensated, if needed, either by means of preemptive digital filtering before the re-sampling action, or by means of an equalizing filter after the downsampling action. In the latter case, the filtering operations must be occasionally suspended at any occurrences of dummy samples.

Typically aliasing effects can be considered negligible for sample rates beneath 10% the maximum sample rate and 8 bit resolution. However, at very high sample rates the overall performance diminishes, suggesting the use of a higher order interpolation filter. Further aspects related to the adoption of alternative filters, are studied in [21] that also presents an implementation of a digital synthesis, based on a 14 nm FinFET GF technology, that shows the feasibility and effectiveness of the approach in terms of hardware resources (less than 400 flip flops) and power consumption (dynamic power less than 1.8 μW/MHz, leakage power less than 1.1 μW). The implementation however evidences a limit for the maximum sample rate that is only slightly above 5 GHz.

## 3. Sample Rate Selection in Multi-Channel Time-Interleaved Architectures

**Figure 2:**
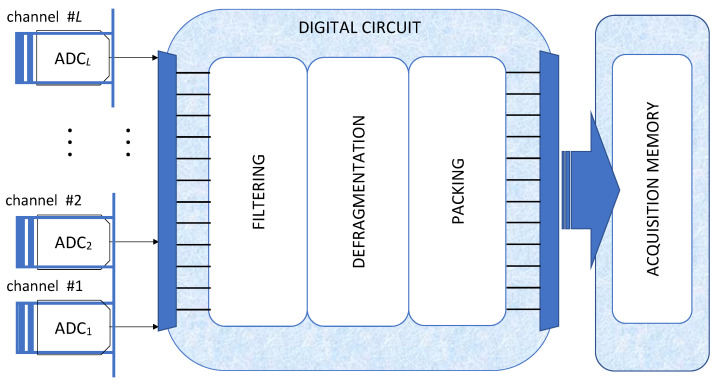
Schematics of the system that enables fine sample rate selection in DSOs.

The proposed solution distributes the processing operations to cope with a stream of bunched samples, each bunch formed with the samples released by the parallel ADCs within the same clock period. In particular, as briefly sketched in Figure 2, it develops through three fundamental stages:

- a filtering stage, where the bunched signal is filtered with a filter characterized by dynamically varying coefficients;
- a defragmentation stage, where the useful samples are separated from the dummy samples;
- a packing stage where a suitable memory management approach assures the acquisition of the useful samples at the selected sample rate.

The processing stages illustrated in the following allow the user to downsample the data stream to *C* times the highest sample rate, where *C* can be within the interval $\left(\frac{1}{2},1\right)$. Fine sample rates can be obtained all over the range by combining the proposed approach with standard decimation with integer values.

### 3.1. Filtering Stage

A DSO with *L* time-interleaved channels, enumerated with l=1,...,L, produces a bunch of *L* samples at each clock tick. If the maximum bunch rate allowed by the system is $f_{ck}$, then the maximum sample rate for the digitized signal, which is obtained by unpacking the bunches, is $Lf_{ck}$. The generic bunch is returned at the discrete time instants *m*, and contains the *L* samples of the digitized signal at the discrete time instants n=mL+l−1, with l=1,...,L.

The sample rate selected by the user is obtained by digitally downsampling the signal at the highest rate, through the linear interpolation method exploited by the single channel architecture. In other terms, the resampled values are determined weighting and summing consecutive samples, which in multi-channel architectures come from adjacent channels. To this end, a couple of *L*-size arrays a(m) and $\bar{a}\left(m\right)$ with components $a=\{a_{1}\left(m\right),...,a_{L}\left(m\right)\}$ and $\bar{a}=\left\{{\bar{a}}_{1}\left(m\right),...,{\bar{a}}_{L}\left(m\right)\right\}=\left\{1-a_{1}\left(m\right),...,1-a_{L}\left(m\right)\right\}$, where $a_{l}\left(m\right)$ is the coefficient corresponding to a given a(n) in a single channel system, namely $a_{l}\left(m\right)=a\left(mL+l\right)$, are required. The arrays are time-varying and must be updated at any new bunch occurrence.

Therefore, in the case of a multi-channel system, one has to use in place of Equation (1):

(3) $$y\left(m\right)=a_{l}\left(m\right)x\left(mL+l-1\right)+{\bar{a}}_{l}\left(m\right)x\left(mL+l\right)$$

Unfortunately, updating cannot be performed through mere linear operations, since, in the presence of dummy values, wrapping is required. In fact, the values of the coefficients could underflow or overflow (0,1); if this happens they must be wrapped around 0 and 1 by respectively adding, in case of underflow, or subtracting, in case of overflow, the unit value. Differently from the single channel implementation, where wrapping is performed at the next clock cycle, for the parallel architecture, it must be performed in the same clock cycle, since the coefficient spoiled by underflow or overflow could be $a_{l}\left(m\right)$, requiring thus wrapping $a_{l+1}\left(m\right)$, which has to be released by the adjacent channel at the same time.

Like the single channel solution, the multichannel one cannot however avoid returning some dummy values, i.e., samples that are estimated using coefficients that undergo wrap operations to fold them into the admissible interval (0, 1); these samples have not significant values and are discarded at the subsequent defragmentation stage. Specifically, the proposed solution works on the individual components of the arrays, and defines the parameter $p_{l}\left(m\right)$ to quantify the number of dummy samples expected in the interval [mL+l,(m+1)L+l]. The parameter $p_{l}\left(m\right)$ can assume either the value P−1 or *P*, which are, respectively, the floor- and ceil-rounded values of the average number of dummy samples, which depends on the value of *C* implied by the sample rate selected by the user, and is given by L(1−C). The generic component of array a(m) at time instant (m+1), namely $a_{l}\left(m+1\right)$, must be obtained by subtracting $L-p_{l}\left(m\right)$ times the lead amount $\frac{1-C}{C}$ and adding the integer $p_{l}\left(m\right)$ for the wrapping operations to the previous value $a_{l}\left(m\right)$:

(4) $$a_{l}\left(m+1\right)=a_{l}\left(m\right)-\left(L-p_{l}\left(m\right)\right)\frac{1-C}{C}+p_{l}\left(m\right)$$

where the parameter $p_{l}\left(m\right)$ by default can be set to P−1 and increased to *P* when $a_{l}\left(m\right)$ is below the threshold:

(5) $$T=\left[L-1-(P-1)\right]\frac{1-C}{C}-\left(P-1\right)$$

which quantifies the decrement that would be applied to $a_{l}\left(m\right)$ in the case that P−1 dummy samples occur in the future L−1 samples. The threshold is defined considering L−1 decrements in order to forecast if an additional decrement produces a negative $a_{l}\left(m+1\right)$ or not. For a coefficient $a_{l}\left(m\right)$ that is below the threshold, the negative value at the next step is not produced if one uses $p_{l}\left(m\right)=P$ in Equation (4), which allows implementing a wrap-around-zero operation.

It is worth noticing that the proposed solution shows that it is not strictly necessary to have the value on the adjacent channel, $a_{l-1}\left(m+1\right)$, for deciding whether a linear decrement or a wrap-around-zero operation is necessary to determine $a_{l}\left(m+1\right)$. In fact, if $a_{l}\left(m\right)$ is a negative or small positive value, it is likely that there is enough room for *P* dummy values in the next *L* values, whereas, in the presence of a higher $a_{l}\left(m\right)$ value the next dummy sample is far enough, so that there is only room for P−1 of them.

It is easy to verify that the generic component of array $\bar{a}$ can be updated at time (m+1) in parallel using:

(6) $${\bar{a}}_{l}\left(m+1\right)=1-a_{l}\left(m\right)+\left(L-p_{l}\left(m\right)\right)\frac{1-C}{C}-p_{l}\left(m\right)$$

The recursive algorithm for updating arrays a and $\bar{a}$ needs a base to start, that consists in the first *L* values of their components, which can be gained through a short run of the serial operations described in Algorithm 1.

**Figure 3:**
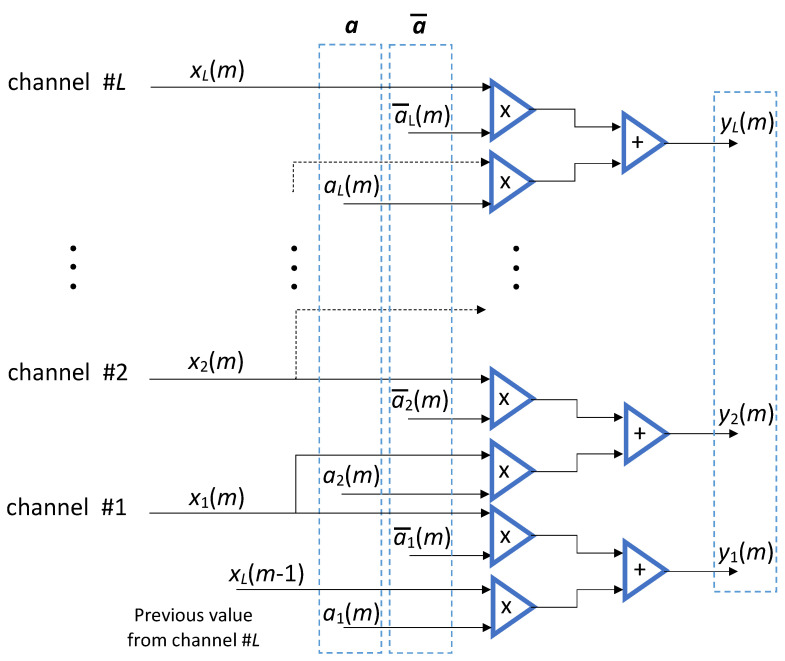
Parallel processing scheme for filtering the bunches produced by the multichannel architecture. The coefficients depend on the time variable *m* and are recalculated at any clock cycle. Useful resampled values are intermingled with dummy values in the output array, y.

At any clock event a bunch of *L* filtered values is finally determined and collected in the array $y=\{y_{1}\left(m\right),...,y_{L}\left(m\right)\}$; to gain y, *L* couples of consecutive samples are needed together with the coefficients in arrays a and $\bar{a}$. The samples come from adjacent channels, namely $x_{l-1}\left(m\right)=x\left(n=mL+l-1\right)$ and $x_{l}\left(m\right)=x\left(n=mL+l\right)$, except for channel 1 that requires an input from the previous bunch, as sketched in Figure 3.

The filtering task returns a couple of *L*-size arrays: the first one is the data array array y, the second one is a boolean array with true and false values distinguishing useful resampled values from dummy values.

### 3.2. Defragmentation Stage

The data array is defragmented by deleting all the dummy values and repositioning the useful values in the lowest cells of the array. The operation is performed by means of an architecture with $\frac{L}{2}$, if *L* is even, or $\frac{L+1}{2}$ if *L* is odd, pipelined blocks: the number of blocks is equal to the maximum number of dummy values that can occur in the acquisition process. Each block includes a data array and a boolean array; the first block accepts as input the arrays produced by the filtering stage. At each step, the data array and the complementary boolean array are transferred to the next block through a bus with switchable lines. The bus configuration is determined by the boolean array, according to the rule that the content of the *l*-th cell is transferred to the new block in cell l−1 when the logic AND on the subarray including the first *l* cells turns false. The described step implies the deletion of the first dummy value encountered scanning bottom-up the data array, and a downshift by one position of all the remaining values. Similarly, the first false value found along the bottom-up direction in the boolean array is deleted. Going through the pipeline, all the dummy values in the input array are deleted and the topmost location, which is freed at each bus switching, is filled with a zero value. Correspondingly, all the false values in the boolean array are deleted and the freed topmost location is filled with a true value.

**Figure 4:**
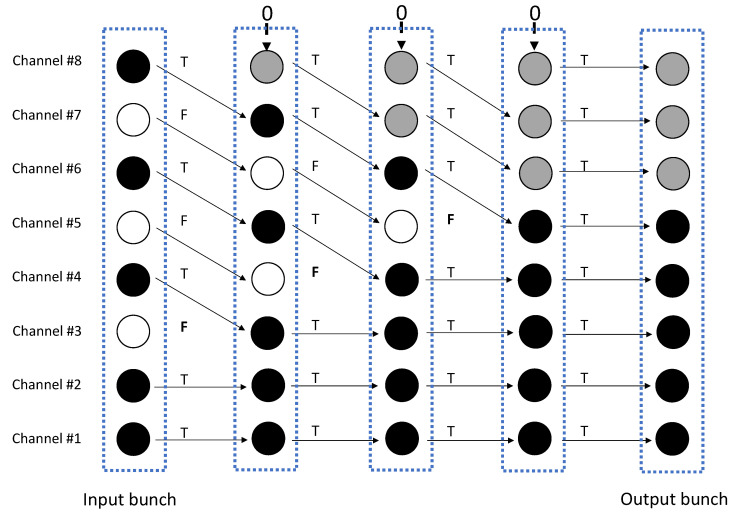
Bunches are defragmented by deleting the dummy samples (white bullets) and retrieving the useful resampled values (black bullets); the topmost positions freed by the data routing operations are filled with zeros (gray bullets).

For the sake of clarity an explaining example is given in Figure 4, where an architecture with L=8 channels is considered. In this case the number of dummy values in the data array can never exceed four, such that a pipeline with four blocks is sufficient. The data array contains three dummy values, shown as white bullets, and five useful values, shown as black bullets. The true or false values of the complementary boolean array are reported aside the same bullets, and permit configuring the lines of the bus for transferring the data in the next block. At the first stage the dummy value deriving from channel 3 is overwritten and the freed topmost location filled with a zero value, represented with a gray bullet. At the second stage the process addresses the value originally derived from channel 5, that has been transferred to the location with index 4 at the previous stage, and is further moved down to the third location in the next block; the third step concerns the third block where the value at index 5, marked with a false boolean, acts on the configuration of the bus for data transfer to the fourth block. Notice that at the last step not one of the bus lines are switched, thus an identical copy of the data array is transferred to the last block. The presence of this further block, which is superfluous in this example, is instead generally necessary to cope with the occurrence of a fourth dummy value, that arises when the value of the factor *C* is near to the lower bound.

The defragmentation stage releases an *L*-size array that contains useful resampled values positioned in the first $L-p_{1}\left(m\right)$ locations, where $p_{1}\left(m\right)$ is the value evaluated on channel l=1 during the filtering stage, and zero values in the remaining locations. The boolean array that has helped at the defragmentation stage is no necessary for the next operations.

### 3.3. Packing Stage

The size of the useful portion of the data array available after defragmentation is not constant during the acquisition process. Since memory management is simpler and more effective in the presence of arrays with constant size, it is convenient grouping the useful values contained in 2 or more consecutive arrays and form constant size arrays to be written in the acquisition memory. To this end, the proposed solution uses the last 3 arrays returned by the defragmentation stage to fill an array with either *L* useful values or *L* zero values, the latter to be regarded as a dummy array. A boolean variable *b* is also allocated to distinguish the array including useful values from the dummy one; only the first has to be saved in the acquisition memory. Actually, both arrays are written in the acquisition memory, but the dummy one is overwritten by the next array. The proposed solution obeys the condition that if a dummy array is written in the last available segment of the acquisition memory, the storage process has to wait for the subsequent array; it is easy to show that a dummy array is always followed by one with useful samples.

**Figure 5:**
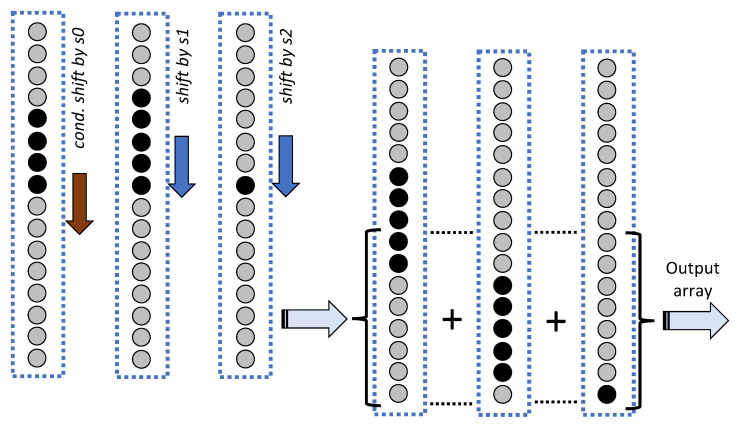
Diagram for the packing operation: the defragmented bunch is copied in the upper half array; as the array goes through a pipelined structure, shifting and zero-filling operations modify the array. Black bullets highlight the useful information, whereas gray bullets represent the zero values that are inserted at the defragmentation or after any shift that frees the topmost locations at the packing stage.

To detail the operation principle of the packing stage, one can imagine of copying the input array in the upper half of a new 2L-size array, that is completed with a lower half with zero values, as shown in Figure 5. The 2L-size array is enqueued in a pipeline architecture, hosting at the generic step 3 arrays, namely the arrays including the bunches at time instants *m*, m−1, and m−2: the first array is at the input of the pipeline in the first position while the third one stands in the third position, i.e., two positions ahead. Each array entering the pipeline contains a number of useful values, which reduces as the array progresses through the pipeline. The number of useful values in the top half part is u0 for the first array, u1 for the array at the intermediate position, and u2 for that at the last position.

At each step parallel shift operations are performed on the 3 arrays: the values in the first array are shifted down by s0 positions, those in the intermediate array by s1 positions, and those in the third array by s2 positions. The downward shifts make room for additional values in the topmost positions, which are all filled with zeros. Adding the half lower portions of the 3 arrays produced by the shifting operations returns the *L*-size array that is written in the acquisition memory. This array is either full of useful values, which represent consecutive samples at the fine sample rate selected by the user, or full of zero values. A zero *L*-size array is forewarned by the occurrence of a false value of the boolean variable *b* at the previous step. In fact, the boolean variable is set false when all the useful values in the intermediate array are necessary to assemble an *L*-size array with useful values; the intermediate array emptied of useful values is found at the next step in the third position.

**Figure 6:**
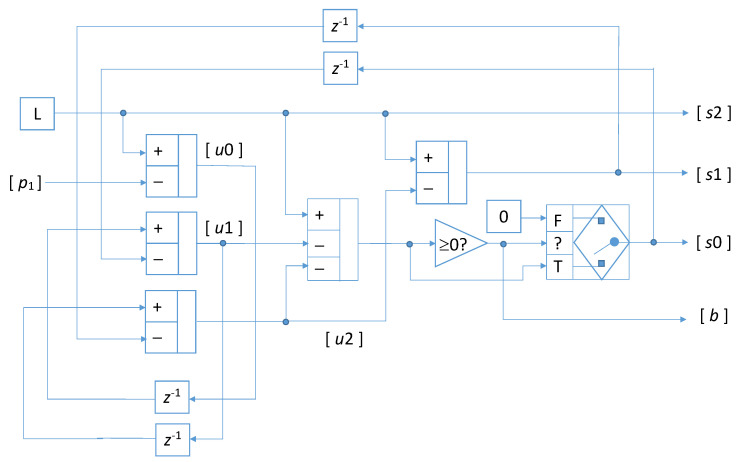
Block diagram illustrating the computations needed to determine the couples of triples {u0, u1, u2} and {s0, s1, s2}; the input to the circuit is $p_{1}$.

The shift amounts s0 and s1 are determined taking into account the current number of useful values available in each array, quantified by the parameters u0, u1 and u2. Actually, it steady holds that s2=L, whereas s1 changes upon the current value of the variable u2 as s1=L−u2, and s0 is determined through a conditional step as either s0=L−u1−u2, or s0=0 whether the first comes out non negative or not. The parameters u0, u1, and u2 are updated according to the self-explaining relations: $u0\left(m\right)=L-p_{1}\left(m\right)$, u1(m)=u0(m−1)−s0(m−1), and u2(m)=u1(m−1)−s1(m−1), where $p_{1}\left(m\right)$ is the number of dummy values in the array that has been just inserted in the pipeline. The logical and algebraic operations that must be performed within each clock cycle are summarized in Figure 6 by means of a block diagram, including adders, comparison operators, conditional selectors, and unitary delay elements: the delay elements are labeled with their discrete transfer function $z^{-1}$, the constant values, *L* and 0 are distinguished by a rectangular frame, variables are instead presented with their names between brackets.

Notice that the parallel operation scheme defined on a restricted set of 3 arrays is sufficient to assemble at least one every other step an output array full of useful values. In fact, in the most critical scenario, where a null array is in the third position of the pipeline and only one residual value is in the intermediate position, the array in the first position still contains CL useful values, and an array with at least CL useful values is incoming in the pipeline. As a consequence, after releasing the null array the total number of values available in the pipeline is 2CL+1, which is greater that *L* for any *C* within $\left(\frac{1}{2},1\right)$.

The main steps performed by the algorithm to packing the data can briefly be summarized in the following bullet point list:

- the 3 arrays are extended with *L* zeros in the bottom part;
- shifts are operated to align the useful values;
- the bottom halves of the arrays are summed and sent to memory;
- the topmost halves are moved to the next step of the pipeline, thus discarding the older half;
- the triples {u0, u1, u2} and {s0, s1, s2} are updated.

### 3.4. Further Remarks

The low-pass effects produced by the interpolation algorithm, can be performed either on the data at the maximum sample rate by means of pre-emptive filtering or on the downsampled data via post filtering. In both cases the filters are conveniently designed using poly-phase design approaches, that inherently take into account that the samples are released in bunches of constant size, equal to the number of channels. In the first case, the pre-emptive filter operates on a data stream characterized by an overall sample rate equal to the maximum rate offered by the system, whereas in the second case the useful data stream made up of the packed data has a sample rate equal to the selected sample rate, and the filtering operations must be suspended at any occurrences of dummy arrays.

Furthermore, time-interleaved channels need calibration to remove offset, gain and time mismatch errors between channels. Designing the digital filters for the streamline calibration requires the knowledge of the frequency responses of the individual channels, which are measured at the manufacturing stage. Standard calibration approaches are implemented in streamline by means of so many digital filters as the number of channels. These filters process the samples released in bunches of constant size; each filter takes in input the sample of the bunch released by the associated channel. The proposed method assumes that calibration is performed before re-sampling. Calibration after re-sampling seems much more difficult, if not unfeasible, because the samples addressed to the acquisition memory, although still released in bunches of constant size, are linear combinations of samples from adjacent channels that have no steady correlation with their native channels.

## 4. Numerical Results and Synthesis of the Circuit

**Figure 7:**
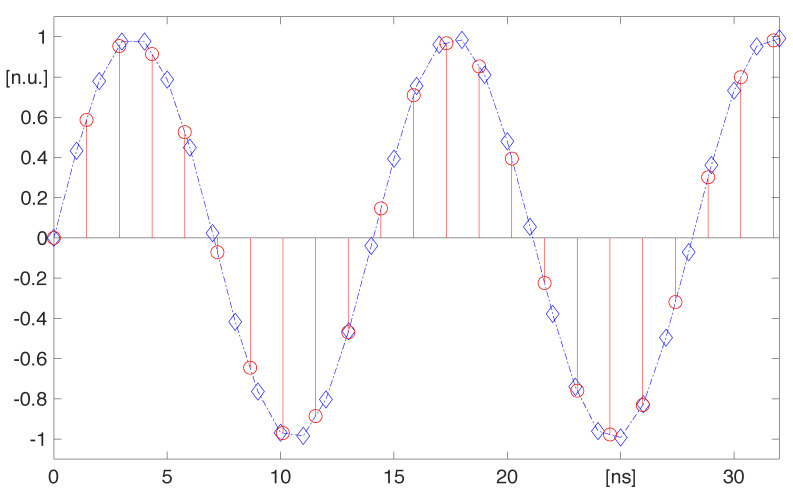
The sinusoidal signal sampled at the 1 GHz (diamond markers) is down-sampled to 693 MHz (circle markers); amplitude values are normalized to the range.

The proposed method is also commented with reference to some numerical data, related to a sinusoidal signal that has been digitized at a sample rate equal to 1 GHz and then re-sampled at 693 MHz, according to the user selection *C* = 0.693, by a multi-channel system with 8 channels. Figure 7 shows both the samples produced at the maximum sample rate with diamond markers, which are interpolated with a dashed line to show the input waveform, and the re-sampled values with stemmed circle markers. The amplitude values are normalized to the range of the converter. By inspecting Figure 7 one can notice that each re-sampled value is amid a couple of consecutive diamond markers, but there are couples of diamond markers that have not re-sampled value in between.

Actually, the 8-channel architecture operates with a slower clock, set to 125 MHz, and releases at each clock an 8-size array x, that collects the samples produced by the 8 channels that operate in time-interleaved mode in the natural sequence, i.e., channel 1 the first, channel 8 the last.

**Figure 8:**
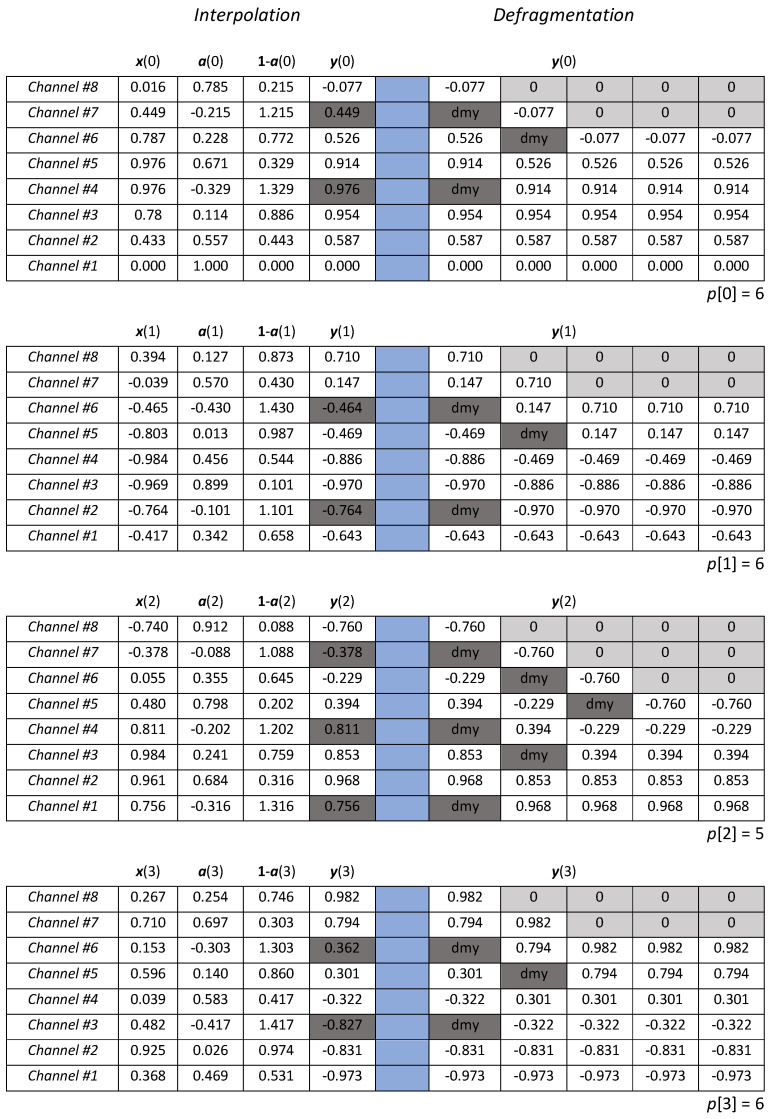
The multi-channel system acquires 8-size arrays, x, that are processed to gain the re-sampled values, y. Sampled and resampled values are enlisted from bottom to top in their columns; two additional columns show the coefficient arrays adopted to perform the interpolation. All the steps needed to attain the defragmentation of the re-sampled data are detailed on the right side.

The data in Figure 7 are also given in Figure 8, where they are organized into 4 tables, related respectively to the 8-size arrays collected by the multi-channel system at the time instants *m* = 0, 1, 2, and 3. These numerical data allow highlighting both the interpolation and defragmentation operations. In particular, the tables contain the input data x, the time-varying coefficients utilized to linearly combine adjacent input values (arrays a and 1-a), and the re-sampled values y. After the interpolation, the array containing the resampled values is defragmented in order to remove the dummy values, as shown on the right side of the table, where the useful values are compacted in the lower portion of the array, and counted by *p*.

**Figure 9:**
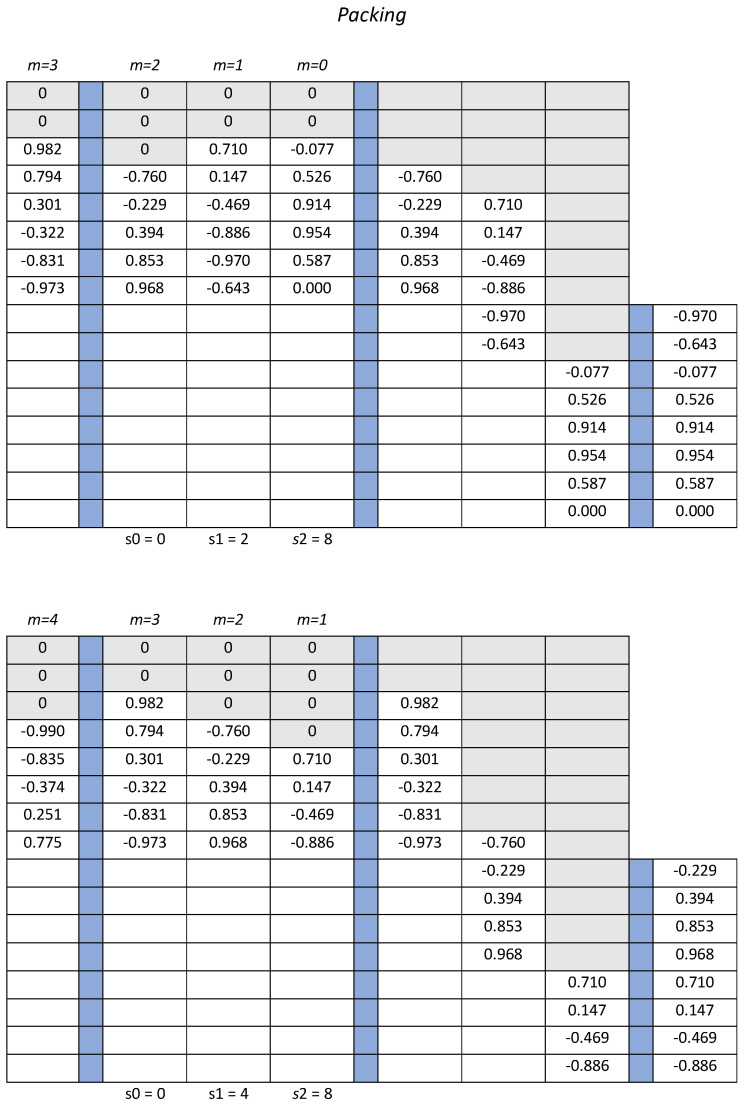
Two subsequent steps of the packing operations are detailed using the re-sampled data given in Figure 8; the 8-size arrays on the right side are the ones stored in the acquisition memory.

Figure 9 shows the numerical data related to the packing operations for the first two processing steps. In particular, the re-sampled and defragmented values are shown in the upper half of the table on the left side, and as moved down by the shifting operations on the right side. The output produced by the packing operation is highlighted with the half-column on the rightmost part of each table.

The proposed solution represents an add-on for the circuitry that interfaces the time-interleaved channels to the acquisition memory. A physical synthesis in a 14 nm FinFET technology, performed by means of Cadence Genus equipped with a commercial standard-cell library provided by Global Foundry for estimating parasitic effects, has been carried out.

Clearly, translating the operation principles described in the aforementioned paragraphs in the design of a digital circuit needs some additional work to step from abstract approaches and face practical issues. However, a detailed description of the layout of the synthesized circuit is beyond the scope of the authors, who just highlight in the following some results featured by the physical synthesis.

The implementation of the add-on circuit for a 64-channels system is described in detail in [21], and demonstrates that:

- the circuit can run at a clock equal to 3.42 GHz, that allows a maximum sample rate equal to 218.88 GHz;
- the silicon area occupation is less than 16,650 μm${}^{2}$, that are divided as 58% for the filtering stage, 27% for the defragmentation stage, and 15 % for the packing stage;
- the dissipated power at the maximum clock frequency is 853 mW, including leakage power equal to 106 μW and dynamic power 249 μW/MHz.

## 5. Conclusions

The issue of selecting the sample rate with fine frequency resolution in digitizers that operate with a fix frequency clock has been addressed. A solution that allows selecting the sample rate as a rational submultiple of the maximum sample rate has been proposed. The proposed solution can improve the functionality range of the time base of digital oscilloscopes that at present is still limited to a few settings that prevent optimal usage of memory resources and coherent sampling operations. The value of the contribution consists in extending the basic principle, already available for digitizers made up of a single channel hosting a monolithic analog-to-digital converter, to the case of digitizers with time-interleaved converters. To this end, the tough issues related to the need of effectively performing complex operations such as (i) filtering of high-rate parallel data streams with time-varying filters, (ii) defragmentation of data arrays to single out useful samples and discard dummy ones, and (iii) data packing to transfer fixed-size data array to the storage memory, have been faced and original solutions presented.

## Data Availability

The data presented in this study are available on request from the corresponding author.
